# Beyond the Posts: Analyzing Breast Implant Illness Discourse With Natural Language Processing and Deep Learning

**DOI:** 10.1093/asj/sjaf047

**Published:** 2025-04-02

**Authors:** Arman J Fijany, Cole A Holan, Anthony E Bishay, Michael J Boctor, Lisandro Montorfano, Ronnie N Mubang, Aparna Vijayasekaran, Jorys Martinez-Jorge, Christin A Harless, Wesley P Thayer, Lauren M Connor, William C Lineaweaver, Elizabeth D Slater

## Abstract

**Background:**

Breast implant illness (BII) is a spectrum of symptoms some people attribute to breast implants. Although causality remains unproven, patient interest has grown significantly. Understanding patient perceptions of BII on social media is crucial because these platforms increasingly influence healthcare decisions.

**Objectives:**

The purpose of this study is to analyze patient perceptions and emotional responses to BII on social media using Robust optimizing Bidirectional Encoder Representations from Transformers, a natural processing model trained on 124 million X posts.

**Methods:**

Posts mentioning BII from 2014 to 2023 were analyzed using 2 natural language processing models: 1 for sentiment (positive/negative) and another for emotions (fear, sadness, anger, disgust, neutral, surprise, and joy). Posts were then classified by their highest scoring emotion. The results were compared over across 2014-2018 and 2019-2023, with correlation analysis (Pearson correlation coefficient) between published implant explantation and augmentation data.

**Results:**

The analysis of 6099 posts over 10 years showed 75.4% were negative, with monthly averages of 50.85 peaking at 213 in March 2019. Fear and neutral emotions dominated, representing 35.9% and 35.6%, respectively. The strongest emotions were neutral and fear, with an average score of 0.293 and 0.286 per post, respectively. Fear scores increased from 0.219 (2014-2018) to 0.303 (2019-2023). Strong positive correlations (*r* > 0.70) existed between annual explantation rates/explantation-to-augmentation ratios and total, negative, neutral, and fear posts.

**Conclusions:**

BII discourse on X peaked in 2019 characterized predominantly by negative sentiment and fear. The strong correlation between fear/negative-based posts and explantation rates suggests social media discourse significantly influences patient decisions regarding breast implant removal.

Breast augmentation with implants is the most performed aesthetic surgical procedure performed in the United States.^1,2^ Since their introduction in the 1960s, breast implants have undergone tremendous scrutiny because of safety concerns.^3^ Fervent attentiveness toward breast implant safety has led to silicone breast implants becoming one of the most tested and scrutinized medical devices. However, since their introduction, there has been a lack of evidence demonstrating causality for physiologic toxicity with their use.^4,5^

Nevertheless, over the past decade, there has been a marked rise in patients with breast implants who present with a constellation of nonspecific symptoms, such as fatigue, brain fog, chronic pain, hair loss, and depression—which they often attribute to breast implant illness (BII),^6,7^ an unofficial medical condition that has gained popularity through social media.^8,9^ Many of these individuals report an improvement in their symptoms after explantation or removal.^10-14^ Furthermore, en bloc capsulectomy—an invasive procedure currently only indicated for breast implant–associated anaplastic large cell lymphoma (BIA-ALCL) and severe cases of capsular contracture—has been increasingly proposed as a solution on social media for women with BII.^15^ These experiences are sometimes shared through social media, which serves as a proxy for quantifying public concern with the condition.

Recent studies have established a rise in internet searches and posts related to BII,^16,17^ as well as published data demonstrating a significant increase in explantation surgeries to remove breast implants.^18^ When comparing published data from 2022 and 2019, there was an ∼10% decrease in breast augmentation volume, whereas implant replacement/removal doubled over that period.^19^ Furthermore, a rising proportion of postmastectomy patients are choosing autologous breast reconstruction over implant-based options.^20^ Overall, it appears that breast implants potentially are losing favorability within the public eye.

Social media platforms are an influential source of medical information, particularly for new and poorly understood disease processes.^8^ These platforms allow many individuals to share their anecdotal experiences on these platforms with others and create support groups.^21^ Recently, there has been a growing impact of social media platforms,^22^ increasing social media usage among the public,^23^ and rising levels of medical mistrust and misinformation.^24^ Unsurprisingly, increased social media use has been positively associated with medical mistrust.^25^ Understanding patient health beliefs and their sources is critical in our media-dominated landscape.

Particularly during the COVID-19 pandemic, social media platforms, such as X (formerly known as Twitter), became a standard tool for disseminating health information.^26^ Users and healthcare providers began sharing their opinions, feelings, and beliefs more often than before. In addition, users on X are usually more candid with their discourse than on other social media platforms. Finally, unlike other social media platforms, X's application programming interface allows for the analysis of public text and data for researchers.^27^

Although there have been previous studies investigating BII social media discourse volume, search engine volume,^17^ and subjective analysis of the discourse on these websites,^9^ there has yet to be an methodical assessment of the sentiment on social media regarding BII and how it correlates to explantation rates. Sentiment analysis is a subsection of natural language processing (NLP) that can assess an individual's stances, feelings, and emotions based on their writing.^28^ Robust optimizing Bidirectional Encoder Representations from Transformers (RoBERTa) is a pretrained deep neural network NLP model trained to assess text sentiment and systematically assign emotions and intensity scores for those emotions.^29^ The model is trained on a large amount of text and is able to decipher words in context bidirectionally—both forwards and backwards. This allows the model to grasp the nuances and intricacies of language, such as sarcasm or wordplay, to accurately determine the intent behind a given text.

Here, we use RoBERTa to analyze posts on the popular social media platform X to assess public sentiment on BII. Additionally, we will compare changes in public sentiment regarding the disease and its history on the platform, paying particular attention to how sentiment changed over this period and the correlation of sentiment to published annual explantation and explantation-to-augmentation rates. Lastly, we will identify common words found in posts that mention BII and perform a thematic analysis on the most popular posts related to BII.

## METHODS

### X Post Inclusion Criteria

The X platform advanced search function was queried month by month for posts over the past decade that included “breast implant illness” by the authors A.J.F. and A.E.B. Posts that were not in English, did not directly mention BII, or mentioned BII in the context of a cancer-related process were excluded. Posts that mentioned BIA-ALCL and BII as separate entities of concern were included. Repetitive posts from the same account were only included as a single post per day. Posts were manually collected into an Excel sheet (Microsoft, Redmond, WA), and each Excel file sheet was organized by month.

### Post Preprocessing and Sentiment Analysis

PyCharm Community Edition 3.4 software is a Python language-based programming environment utilized for our data and sentiment analysis functions.^30^ To prevent words in user names and website names from being included in our emotional analysis, posts were preprocessed by removing usernames and websites by defining every portion of the text that began with “@” as “@user” and “http” for every text with “http” in it.

Posts were then inserted into 2 different models for sentiment analysis. The first model, created by Loureiro et al, analyzed the text and assigned “positive,” “negative,” and “neutral” scores from 0 to 1, which were ordered from highest to lowest.^31^ Modifications were made to this model to classify each post as either “negative” or “positive” based on the higher score.

The second model was built to classify emotion in text based on Ekman's 6 fundamental emotions—fear, sadness, anger, disgust, surprise, and joy—as well as a neutral class.^32,33^ The second model also assigned a score for each post about these 7 emotions from 0 to 1. In summary, each post was categorized as “positive” or “negative” in the first model and the highest scoring emotion from the second model (Table 1). Posts were then counted by category and mean weighted group scores were calculated. Examples of high-scoring posts for each output are shown in Table 2.

**Table 1.** Total Annual Posts by Bimodal Sentiment (Model 1) and Emotional Classification (Model 2)

	Model 1		Model 2							
Year	Negative	Positive	Fear	Joy	Disgust	Anger	Sadness	Surprise	Neutral	Total
2023	574	130	198	30	54	17	78	41	285	704
2022	721	204	315	42	48	19	127	46	328	925
2021	532	182	247	32	45	23	83	43	241	714
2020	801	254	339	53	40	17	170	53	382	1055
2019	1078	431	530	104	55	32	174	94	520	1509
2018	477	145	208	41	21	14	68	21	271	622
2017	287	99	286	35	9	2	119	4	100	386
2016	108	50	38	11	10	0	37	8	58	158
2015	20	2	9	1	0	7	2	1	2	22
2014	2	2	1	0	0	0	0	0	3	4
Total	4600	1499	2171	349	282	131	858	311	2190	6099

**Table 2.** Correlation Coefficients Between Sentiment Classification (Fear, Negative, Neutral, and Total Posts) With the Implant Explantation Rate

Group	Post	Model 1	Model 2
Positive	Definitely your choice and you would look great either way I would say look into breast implant illness first so you can make an informed decision!	Positive 0.92	Neutral 0.78
Negative	NO!!!!! I beg you DONT. If you are serious please look into BII aka Breast Implant Illness. It is a devastating condition and can be fatal	Negative 0.94	Sadness 0.61
Neutral	Breast Implant Illness Stories: The Journey of a BII Patient If you have any questions pertaining to Bii Call (561) 823-1179 to schedule a consultation with Dr. Barr	Neutral 0.93	Neutral 0.65
Fear	Breast implant illness terrifies Me	Negative 0.90	Fear 0.98
Sadness	She suffered for 6 years—it was Breast Implant Illness	Neutral 0.55	Sadness 0.95
Anger	breast implant illness & IUD illness is real Woman begging them to remove a foreign objected from their body and REFUSING because lab results are normal, but the pain & symptoms are present is absurd. When a patient wants something out of their body they should take it out!	Negative 0.86	Anger 0.85
Disgust	Really liked that you talked about aluminium etc in body products. However, I am not sure if this is the case, but if you have had a breast augmentation you should really look up Breast implant illness, I am sure you will find it very disturbing since you do care a lot about this	Neutral 0.44	Disgust 0.86
Surprise	I was listening to something this morning that said she had Breast implant illness. Apparently this is due to having super old implantsWonder if this is the case?	Neutral 0.55	Surprise 0.95
Joy	the fact that breast implant illness is being talked about soooo much these days makes me happy . women need to be more educated on the foreign objects they’re putting in their bodies	Positive 0.83	Joy 0.98

### Radar Plot Generation

Average annual scores for Ekman's 6 emotions were input into PyCharm for radar plot generation using the plotly library. Radar plots were generated yearly from 2016 to 2023, a 5-year comparison between 2014-2018 and 2019-2023, and a total cohort radar plot.

### Word Cloud Generation

PyCharm software was used again to generate word clouds—graphic illustrations of specific words most frequently found in multiple pieces of text. Word clouds were generated for 5-year periods (Supplemental Figure 1 for 2014-2018 and Supplemental Figure 2 for 2019-2023) and the entire decade cohort of X posts (Supplemental Figure 3). Stop words, such as “breast,” “implant,” “illness,” “at,” “I,” “the,” and other common nondescriptive words, were excluded from the analysis.

### Correlation to Implant Procedure Data

Augmentation and explantation data were pulled from published annual reports from The Aesthetic Society.^18^ Each year's annual augmentation and augmentation data were pulled and organized into a table. Total, negative, neutral, and fear posts were correlated to the annual explantation and explantation-to-augmentation ratio. Positive correlation was classified by the Pearson correlation coefficient (*r*) with the following groups: “very strong” (*r* = 0.7-1), “moderate” (*r* = 0.4-0.69), “moderate” (*r* = 0.3-0.39), “weak” (*r* = 0.20-0.29), and “negligible” (*r* = 0.00-0.19), as previously outlined in Akoglu.^34^  *P*-values were determined by referring to a *t*-distribution with *n* − 2 degrees of freedom, which is the most commonly used methodology to assess significance for Pearson correlation coefficient.^35^ Statistical significance was determined with a *P*-value of <.05.

## RESULTS

Overall, 6099 posts from X were included in our cross-sectional analysis (Table 1). Of these, most were classified as negative in our first model (75.4%), and approximately a quarter were positive (24.6%). In the second model, the most classified emotions were neutral (35.9%) and fear (35.6%). These emotions were followed by sadness (14.1%), joy (5.7%), surprise (5.1%), disgust (4.6%), and anger (2.1%). The first post that mentioned BII was posted on June 8, 2014. Posts that mentioned BII peaked in March 2019, with 213 posts that month. In 2019, 1509 posts referred to BII. Recently, there were 704 posts in 2023.

**Figure 1.**
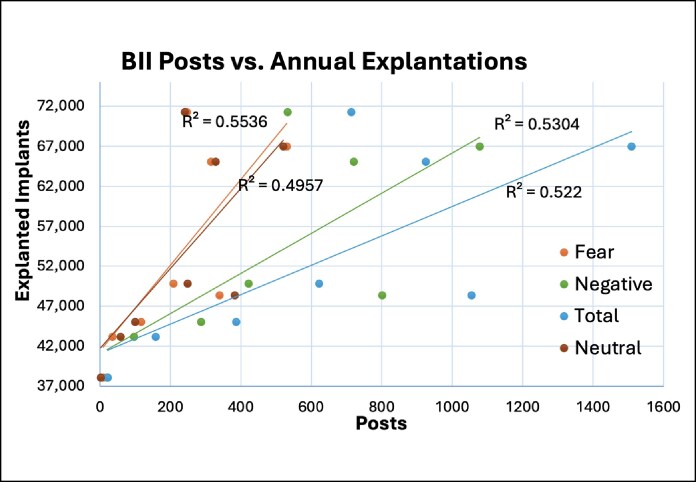
Relationship between BII-related posts vs the annual number of implant explantations. *R*^2^ denotes the coefficient of determination. BII, breast implant illness.

**Figure 2.**
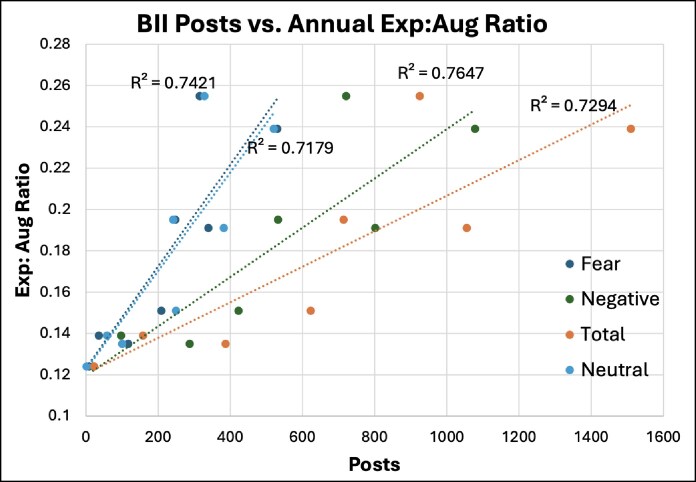
Relationship between BII-related posts and the ratio of explantation to augmentation per year. *R*^2^ denotes the coefficient of determination. BII, breast implant illness.

Augmentations peaked in 2021, with a total of 364,753 recorded procedures (Table 3).^36^ The highest recorded annual explantation count was 71,284 in 2021 (Figure 1). Pearson correlation coefficients for fear, total, negative, and neutral posts all displayed a “very strong” (*r* > 0.70) positive correlation to the annual explantation count and explantation-to-augmentation ratios (Figure 2). This correlation was significant (*P* < .05) for all groups except for neutral posts to the annual explantation-to-augmentation ratio (Table 4).

**Table 3.** Annual Breast Implant Augmentations and Explantations, With Corresponding Social Media BII Post Counts^a^

Year	Aug	Exp	Fear	Total	Negative	Neutral
2023	NR	NR	198	704	574	285
2022	255,200	131,758	315	925	721	328
2020-2021^a^	364,753	147,684	586	1769	1333	323
2019	280,692	110,286	530	1509	1078	520
2018	329,914	114,246	208	622	422	249
2017	333,392	45,024	117	386	287	100
2016	310,444	43,181	35	158	97	58
2015	305,856	38,071	9	22	20	2
2014	286,694	NR	1	4	2	3

Aug, augmentation; Exp, explantation.

^a^Data provided from The Aesthetic Society Annual Procedural Statistics.^18^

**Table 4.** Correlation Coefficients Between Sentiment Classification (Fear, Negative, Neutral, and Total Posts) With the Implant Explantation Rate

Pearson correlation coefficient (*r*)				
Post type	Fear	Negative	Total	Neutral
Exp	0.744^a^	0.728^a^	0.722^a^	0.704
Exp:Aug	0.862^a^	0.875^a^	0.854^a^	0.847^a^

Aug, augmentation; Exp, explantation.

^a^Statistical significance at *P* < .05.

**Figure 3.**
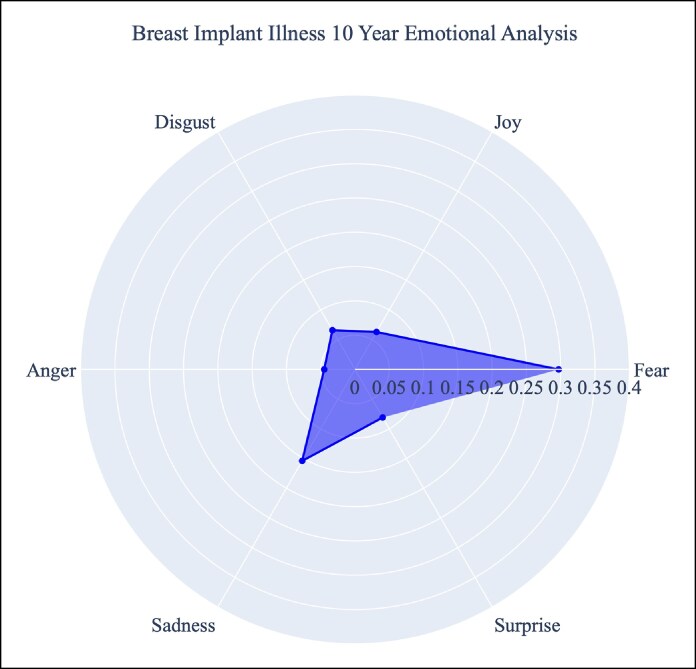
Radar plot with the distribution of BII-related emotional sentiment over a 10-year period. BII, breast implant illness.

**Figure 4.**
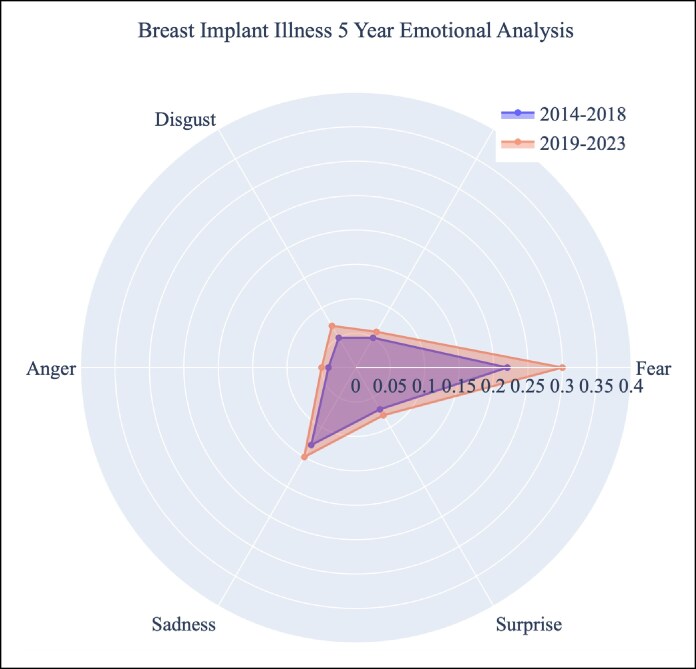
Radar plot with the distribution of BII-related emotional sentiment over 5-year periods. BII, breast implant illness.

The highest average emotion scores were neutral (0.293), fear (0.286), and sadness (0.144) over the 10 years (Table 5, Figure 3). The lowest average emotion scores were anger (0.045), joy (0.060), disgust (0.066), and surprise (0.081). Every emotion's average score grew in intensity over the past 5 years compared with the previous 5-year period (Figure 4). Of these emotions, fear had the most significant growth (+0.084). From 2014 to 2018, the average fear emotion score was 0.219. Over the next 5-year period (2019-2023), the average fear emotion score was 0.303. Within posts that mention BII, common words found within the text include: “symptom,” “real,” “women,” “health,” “silicone,” “year,” “issue,” “removed,” “problem,” “risk,” “bad,” “scare,” “common,” “disease,” and “immune” (Supplemental Figures 1-3).

**Table 5.** Annual Weighted Average Emotion Scores for BII-Related Social Media Posts Over Time

Time period	2023	2022	2021	2020	2019	2018	2017	2016	2015	2014	2014-2018	2019-2023	2014-2023
Neutral	0.33	0.30	0.28	0.30	0.28	0.31	0.22	0.33	0.14	0.25	0.28	0.30	0.29
Fear	0.29	0.31	0.30	0.30	0.31	0.31	0.29	0.20	0.31	0.15	0.22	0.30	0.29
Joy	0.06	0.05	0.05	0.06	0.07	0.07	0.10	0.07	0.05	0.05	0.05	0.06	0.06
Disgust	0.11	0.08	0.08	0.06	0.05	0.05	0.04	0.08	0.03	0.09	0.05	0.07	0.07
Anger	0.05	0.04	0.05	0.05	0.04	0.06	0.03	0.03	0.30	0.01	0.04	0.05	0.05
Sadness	0.14	0.15	0.15	0.16	0.15	0.13	0.28	0.22	0.15	0.12	0.13	0.15	0.14
Surprise	0.08	0.07	0.09	0.08	0.09	0.08	0.05	0.09	0.03	0.02	0.07	0.08	0.08

BII, breast implant illness.

## DISCUSSION

In the 1980s, silicone implants gained significant media attention because of similar nonspecific autoimmune and rheumatological symptoms. The amplification of small case series and individual reports on media sources of debilitating self-reported prodromes after silicone implant placement resulted in an FDA moratorium on silicone implants in 1992.^37^ Several studies were then performed in the 1990s, which discovered no increased risk for autoimmune-like diseases,^38^ no increased antinuclear antibodies,^39^ and no increase of adjuvant disease in those individuals with ruptured implants,^40^ and no increased association with connective tissue disorders.^41,42^ These studies, and a few others, were instrumental in the later lifting of the moratorium on silicone implants in 2006.^37^

Recently, there has been a resurgence in concerns with breast implant safety and a corresponding increase in patients who request to have their implants removed. Many individuals have associated this resurrection with the media amplification of BII.^8,9,43^ A 2021 survey-based study conducted on a BII advocacy group on Facebook found that most survey respondents (56%) were first exposed to BII from social media, with only 2% reporting that their first description of BII was from a plastic surgeon.^44^ Moreover, the debate between a causal vs correlative association between silicone breast implants and BII continues. Recently, some have argued that there is a causal association between an autoimmune-like disease and silicone breast implants using the Bradford–Hill criteria.^6^ Others have used the same criteria to rule out causality.^45,46^

**Figure 5.**
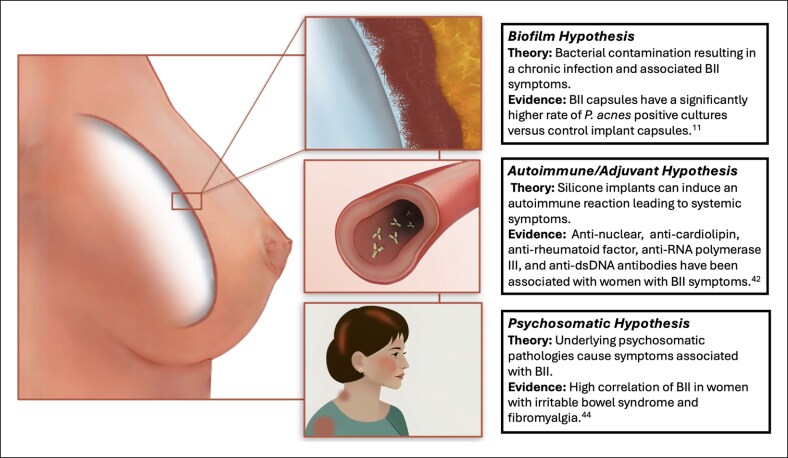
Three predominant BII hypotheses: biofilm, autoimmune/adjuvant, and psychosomatic. BII, breast implant illness.

Apart from causality, there is significant evidence for a positive association between women with silicone implants and autoimmune conditions.^47^ There are 3 predominant hypotheses for the association between silicone implants and systemic symptoms—biofilm, autoimmune, and psychosomatic (Figure 5). In a large cross-sectional study, there were significantly increased odds of being diagnosed with an autoimmune or rheumatological disorder with breast implants.^48^ In another recent survey, the authors compared women with BII who underwent explantation to women with breast implants without BII and demonstrated an over 20-fold increase in the self-reported rate of fibromyalgia and an over 4-fold increase in irritable bowel syndrome in the explanted BII cohort.^49^ Implants from patients with self-reported BII demonstrated a high rate of chronic infection with *Propionibacterium acnes*.^11^ Conversely, the authors of the follow-up studies have shown that the presence of bacteria is not associated with BII symptoms.^50^ For those people who chose to undergo explantation, there is ample evidence for symptom improvement postimplant removal.^51-53^ However, there are also studies where the authors demonstrate no improvement in symptoms with capsulectomy and symptom recurrence 6 to 12 months postexplantation and capsulectomy.^54,55^

In June 2024, the Breast Surgery Collaborative Community delivered a consensus statement that stated that an en bloc capsulectomy is not indicated for women with suspected BII.^56^ Because of the lack of scientific evidence for any benefit of en bloc capsulectomy, coupled with the increased risks and costs of the procedure, the majority of plastic surgeons have advised against the procedure. Still, there is a growing demand for the procedure, which has been shown to be driven at least in part by social media and surgeons who seek to benefit financially from performing the procedure.^15^

Humans have been described as “emotional creatures,” and studies have shown that emotions can guide the way humans think and act.^57,58^ In this study, we investigate the psychosomatic origins of systemic symptoms associated with breast implants by analyzing the emotional discourse surrounding BII on social media platforms. We specifically evaluate how these emotional sentiments, as captured through NLP sentiment analysis, influence patients’ decisions to undergo breast explantation. Previous studies have shown that RoBERTa has >90% accuracy classifying sentiment on posts from X's social media platform.^59^

In this study, we discovered that emotions around BII were initially negative, then gradually shifted toward a heightened emotional expression of fear and sadness, indicating a deepening concern within the online community. Temporal analysis revealed pivotal moments, such as the first X mention in 2014 and the peak in March 2019, reflecting intensified public engagement with the topic of BII over the past decade. Likewise, in this study, we uncovered a strong correlation between emotional sentiment on X and subsequent trends in surgical procedures related to breast implants, highlighting the influence of social media discourse on healthcare practices. There is also a documented increase of depression, anxiety, and poor sleep patterns—all symptoms associated with BII—with social media use.^60^

A major finding was the prevalence of negative sentiments, highlighted by emotions of mostly fear, followed by sadness, indicative of widespread apprehension and uncertainty regarding BII among X users. Notably, the intensity of all emotions, particularly fear, increased from 2014 to 2023. This trend suggests a deepening concern within the online community, reflecting a heightened awareness and emotional resonance with BII. Despite the absence of concrete causal evidence, it is clear from the present findings that public sentiment evolves independently of factual scientific evidence.^61^ Moreover, exploring temporal trends highlighted pivotal moments, such as the peak in X posts in March 2019, which served as markers of intensified public engagement and discourse surrounding BII. These temporal peaks not only underscored shifts in emotional sentiment but also paralleled the increasing presence of BII in the media.^62,63^ This suggests that as the conversation around BII evolved, so did the emotional responses, indicating the topic's growing impact and relevance in online discussions.

There are multiple limitations to our study that are worth noting. First, there is an inherent selection bias with identifying posts related to BII on X, because those who experience BII symptoms are more likely to share their experiences than those who are asymptomatic. This can ultimately lead to an overrepresentation of negative experiences within our dataset. Additionally, the data were pulled from the social media platform X, which is a large but not representative sample of the general population.^64^ Moreover, using NLP has limitations as well. There is a documented weakness in NLP-based models like RoBERTa for understanding sarcasm or irony. However, out of the NLP models used today, RoBERTa has been shown to perform the best in terms of understanding sarcastic or indirect language.^65^ Another limitation was the inability to identify robot accounts. To reduce the effects of robot accounts, we chose to set a limit of a single post per account per day and excluded posts that were repeated by the same account. We also did not account for the influence and reach of each account and post, and a more thorough analysis would perhaps give additional weight to accounts with large followings or posts with a significant number of interactions on the platform. There are also limitations to the explantation data we used for our correlation analysis. The source only includes reported explantations, and it cannot be assumed that all the explantations were because of fear of breast implant illness. Many of the explantated implants were likely removed because of the FDA recall of macrotextured implants because of a risk of BIA-ALCL, capsular contracture, dissatisfaction with breast appearance, or potentially other factors unrelated to BII.^66,67^

Although we recognize the many limitations to our study, we believe that our findings outline the possible role of social media's influence in public perceptions of BII using objective methods. The authors of previous studies have described the influence of social media on BII perceptions, primarily by analyzing postvolume over time.^7,9,14,15,17^ However, to this point, none have used impartial deep-learning models to systematically score the content and sentiment of BII social media posts. Our study provides a novel, data-driven approach to understanding the discourse on social media regarding BII, thereby providing a more comprehensive assessment of how social media content can change over time. These findings highlight the need for additional research into the influence of social and patient decision-making, as well as social media's impact on clinical practices, regulatory policies, and postmarket safety monitoring of medical devices.

The current study revealed a very strong correlation between fear and negative emotional sentiment expressed on X and subsequent trends in annual explantation procedures and explantation-to-augmentation ratios. This finding suggests a positive association between public perception of BII and medical decision-making, highlighting the potential influence of social media discourse on healthcare practices. The impact of social media on healthcare decision-making has been previously examined because of its effects during the COVID-19 pandemic.^68-70^ Importantly, although social media has allowed for more practical communication and large-scale dissemination of information, it has heightened certain risks, such as misinformation and violation of privacy rights.^71^ Recognizing the implications of these findings, healthcare practitioners must adeptly steer conversations surrounding BII, guaranteeing that patients are equipped to make enlightened choices regarding their health and welfare. By acknowledging the emotional undercurrents of BII discourse, clinicians can cultivate transparent exchanges and offer holistic assistance to those individuals impacted by BII, thereby advancing superior patient outcomes and contentment. This correlation underscores the need for further research into the impact of social media discourse on healthcare decision-making and patient outcomes.

### Implications

Armed with insights from the current study, surgeons can understand the correlation between social media discourse and medical decision-making. This underscores the need for continued research into the influence of online sentiments on healthcare practices. Although patients may request removal of their implants or even an en bloc capsulectomy for symptoms associated with BII, it is the duty of the surgeons to discuss the risks and lack of scientific benefits to these procedures.

## CONCLUSIONS

The authors of the current study illuminate the evolving discourse surrounding BII, revealing the prominence of negative sentiments with increasing emotions of fear and sadness, reflecting deepening concern within the online community. Temporal analysis pinpointed pivotal moments, such as the peak in X mentions in March 2019, indicative of intensified public engagement with BII. Additionally, the strong correlation between emotional sentiment on X and subsequent trends in explantation procedures underscores the potential influence of social media discourse on healthcare practices, emphasizing the necessity for ongoing dialogue and research initiatives to promote informed decision-making and comprehensive patient care.

## Supplemental Material

This article contains supplemental material located online at https://doi.org/10.1093/asj/sjaf047.

## Supplementary Material

sjaf047_Supplementary_Data
