# Supplementary figures and images for: Beyond the Posts: Analyzing Breast Implant Illness Discourse With Natural Language Processing and Deep Learning
Source: Aesthet Surg J. 2025 Apr 2;45(7):745–52. doi: 10.1093/asj/sjaf047 (PMC12168447; doi:10.1093/asj/sjaf047)

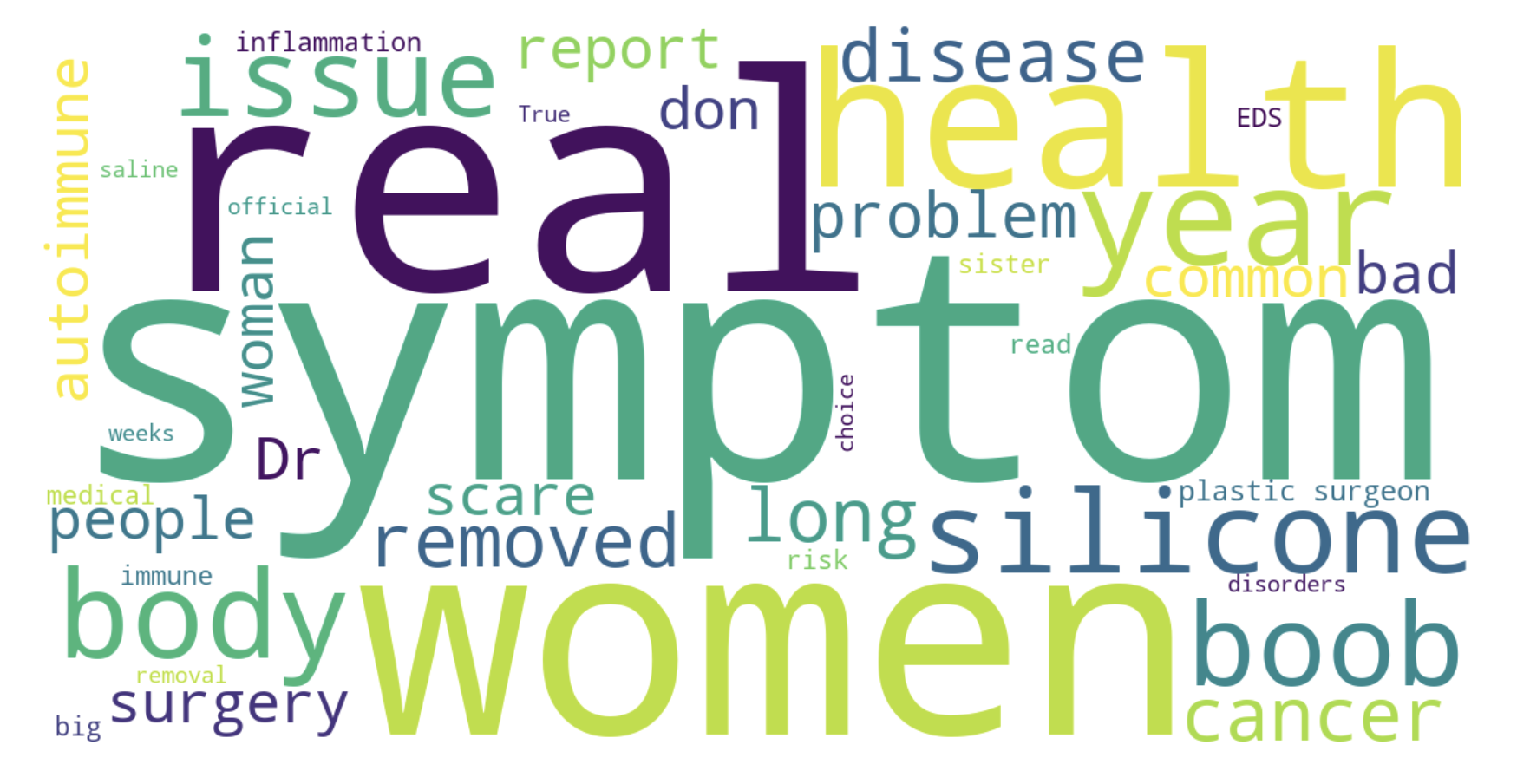

Supplement: sjaf047_Supplementary_Data [file sjaf047_supplementary_data.zip › Supp Figure 1.png]

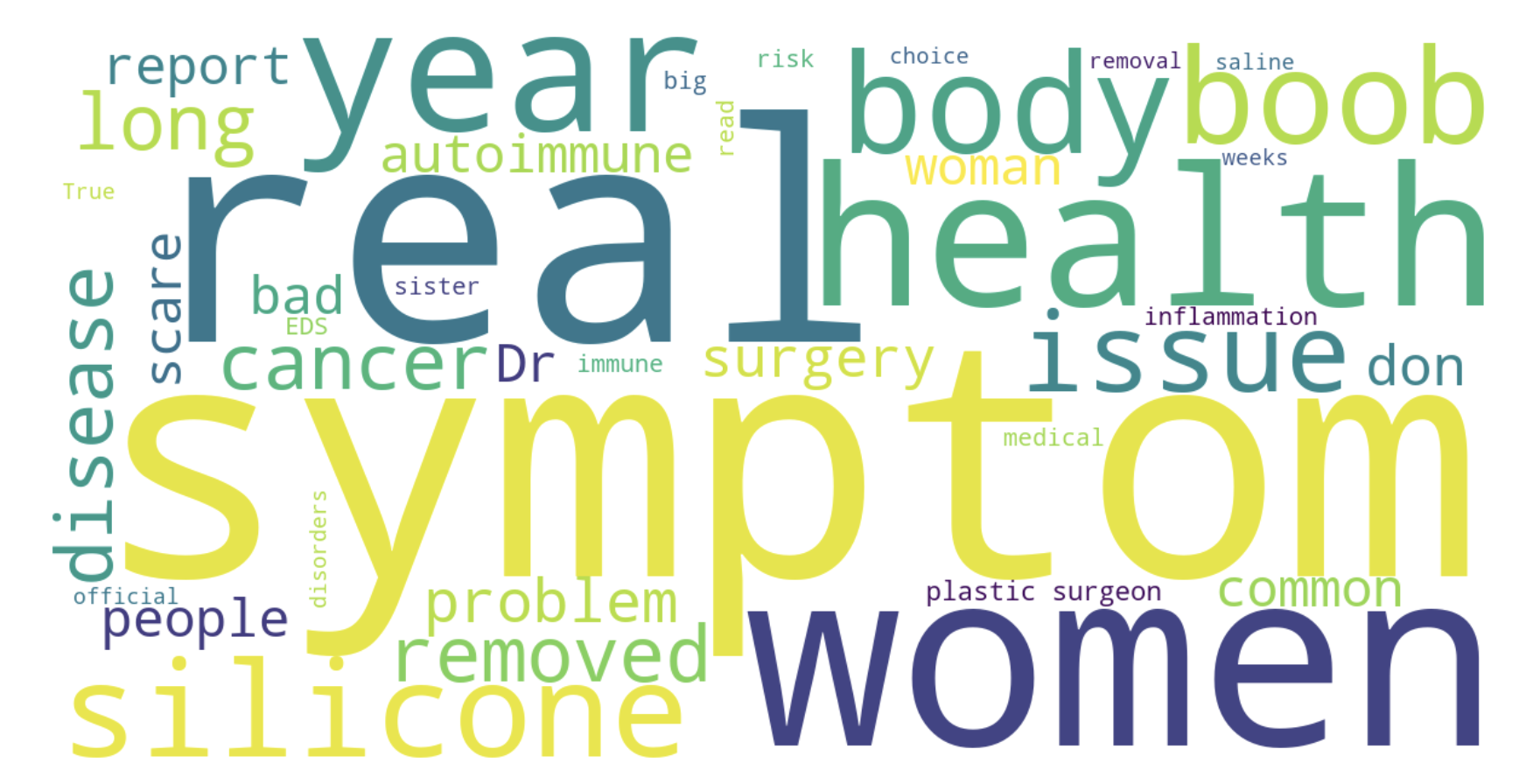

Supplement: sjaf047_Supplementary_Data [file sjaf047_supplementary_data.zip › Supp Figure 2.png]

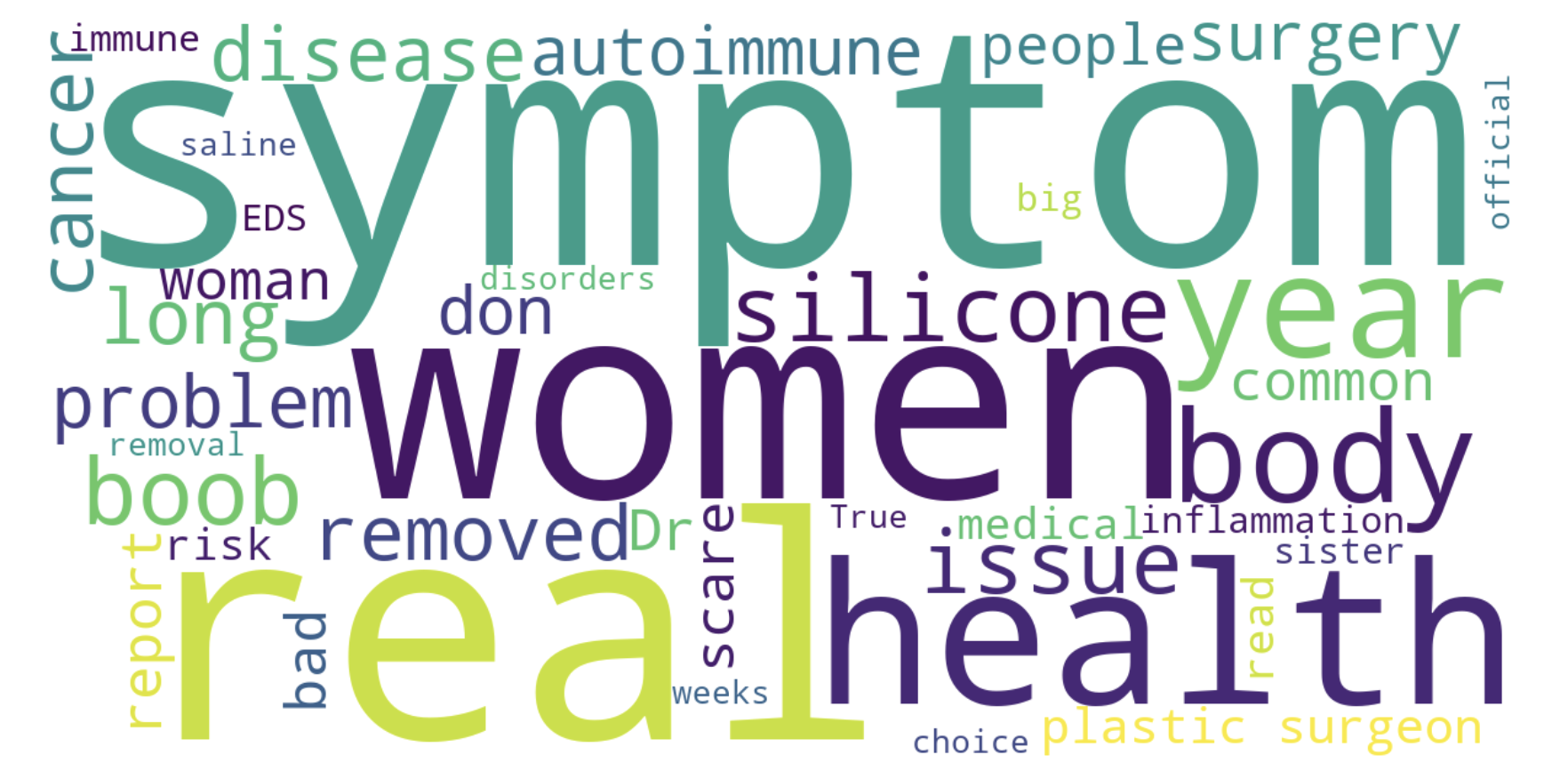

Supplement: sjaf047_Supplementary_Data [file sjaf047_supplementary_data.zip › Supp Figure 3.png]
